# Adaptive Social Factors and Precompetitive Anxiety in Elite Sport

**DOI:** 10.3389/fpsyg.2021.651169

**Published:** 2021-04-15

**Authors:** Heriberto Antonio Pineda-Espejel, Edgar Alarcón, Raquel Morquecho-Sánchez, Verónica Morales-Sánchez, Erika Gadea-Cavazos

**Affiliations:** ^1^Facultad de Deportes, Universidad Autónoma de Baja California, Mexicali, Mexico; ^2^Facultad de Organización Deportiva, Autonomous University of Nuevo León, San Nicolás de los Garza, Mexico; ^3^Facultad de Psicología, Universidad de Málaga, Málaga, Spain

**Keywords:** motivation, sport, instrumental study, psychological need, contextual level

## Abstract

Grounded in achievement goal theory and self-determination theory, the aim of this study was to analyze the motivational determinants of precompetitive anxiety in the sports context, considering the horizontal motivational sequence: adaptive social factors (task-involving climate, autonomy support style), competence need, types of motivation (autonomous motivation, controlled motivation, amotivation), and consequences (precompetitive anxiety and self-confidence). This study was also conducted in order to analyze the mediating role of the need for competition and motivational regulations on social factors and consequences. The sample consisted of 217 athletes of both sexes engaged in elite sport, who answered a series of questionnaires to measure study variables to develop an analysis of the structural equation model. The results showed that both the task-involving climate and autonomy support were associated with competence need, and competence need was associated with autonomous motivation in a positive way and with controlled motivation and amotivation in a negative way. On the other hand, autonomous motivation was positively associated with self-confidence, while amotivation was positively related to somatic and cognitive anxiety before a competition. Furthermore, there was a total mediation of competence need and autonomous motivation between task climate and self-confidence. In conclusion, these social factors favor self-confidence, and besides, these climates disfavor anxiety before a sport competition.

## Introduction

The achievement goal theory (AGT; Nicholls, 1984, 1989) and the self-determination theory (SDT; Deci and Ryan, 1985; Ryan and Deci, 2000) have been useful to explain psychological processes that mediate between social context and the motivational, affective, and behavioral processes of athletes. The SDT takes into consideration some aspects of the AGT, according to some characteristics of the social context created by significant others that influence people's motivation (Vallerand and Losier, 1999).

The AGT conceives the motivational climate as an environment constituted by implicit and explicit signals from significant others, which represent patterns of success (Ames, 1992). Two types are distinguished: task-involving climate, where people perceive that they are rewarded for personal improvement and learning; and ego involving climate, in which people perceive that only the best performers are valued (Ames, 1992). The first has more positive consequences than the second (Ames, 1992). In general, the perceptions of a task-involving climate are associated with more adaptive cognitive and affective patterns.

For its part, the AGT describes the perceiving supportive interpersonal behaviors and thwarting interpersonal behaviors (Ryan and Deci, 2017), for example autonomy support (i.e., self-initiated effort, volition, freedom of choice, and presentation of alternatives), competence support (i.e., positive feedback related to a specific task), relatedness support (i.e., demonstration of emotional support), autonomy thwarting (i.e., enforcement, exerts pressure), competence thwarting (i.e., expression of behaviors that emphasize guilt and doubt), and relatedness thwarting (i.e., perception of behaviors of rejection). When perceiving supportive interpersonal behaviors, people perform more self-determined actions, compared with thwarting interpersonal behaviors (Rocchi et al., 2016).

The SDT postulates that the motivational context is related to people motivation *via* the psychological needs satisfaction (Reinboth et al., 2004) and that the type of motivation experienced by people will have an impact on the emotional, cognitive, and behavioral state (Ryan and Deci, 2000; Gagné and Deci, 2005).

In this respect, the SDT assumes that people are born with three basic psychological needs: competence, which concerns the desire to be effective in interacting with the environment and expressing one's capabilities (Deci, 1975); autonomy, which refers to feelings that one is the origin and source of one's own action; and relatedness, which are the feelings of being related to others. Regarding motivation, SDT behavior is directed by motivation regulations that vary in the levels of self-determination. These motivation regulations are on a continuum ranging from those that are more autonomous to more controlled (Deci and Ryan, 1985; Ryan and Deci, 2000). When individuals feel that their behavior is internally regulated from within, they experience autonomous motivation, a desire to act based on interest in, enjoyment of, or placing value on the work or the behavior itself (Gagné and Deci, 2005). When individuals feel that their behavior is externally regulated by outside forces, such as other people or rewards and punishments, they experience controlled motivation, a desire to act based on a sense of pressure and obligation (Ryan and Deci, 2000). Thus, external regulation can be more controlled, when it is supported by external control (extrinsic regulation) and internal contingencies (introjected regulation), while it becomes more autonomous when it is directed by an instrumental value (identified regulation) or by personal values (integrated regulation). SDT also recognizes that amotivation is characterized by low or lack of motivation (Ryan, 1995).

### Supportive Behaviors, Psychological Needs, and Motivation

Ryan and Deci (2000) suggested that if the social context promotes the psychological needs satisfaction, then the forms of motivation will be as self-determined as possible. It has been shown that autonomy support favors the most self-determined motivation, because it contributes to the psychological needs satisfaction (e.g., Amorose and Anderson-Butcher, 2007; Vallerand, 2007). This has been supported in the sports context in works such as those by Álvarez et al. (2009), who found that autonomy support predicts the psychological needs satisfaction (measured through an index), and this predicts self-determined motivation (measured through an index). In addition, Pope and Wilson (2012) showed that autonomy support was positively related to competence and autonomy, and the need for competence did so positively, both with controlled regulations (i.e., external and introjected) and with autonomous regulations (i.e., identified and intrinsic), and negatively with amotivation. Similar results were obtained by Isoard-Gautheur et al. (2012), with the difference that the need for competence predicted the most controlled regulations and the identified regulation. This was replicated by Balaguer et al. (2012). Recently, Rodrigues et al. (2019) showed that supportive behaviors were positively related to the satisfaction of psychological needs, and these were also positively associated with both autonomous motivation and controlled motivation.

### Task Climate, Psychological Needs, and Motivation

Analyzing the social context based on the more adaptive motivational climate theorized by the AGT (i.e., task climate) and its association with the motivational regulations and psychological needs proposed by the SDT, studies in the sports context show that athletes who perceive a task climate report a high competence need and that this is associated with self-determined motivation (measured with an index) (Sarrazin et al., 2002). Ommundsen et al. (2006) showed that a task-involving climate is related to competence perception. Likewise, Ommundsen et al. (2010) demonstrated that a task climate predicts intrinsic motivation through competence and autonomy needs. Other studies showed that a task climate positively predicts each of the psychological needs, and these predict intrinsic motivation (Almagro et al., 2011).

Despite the aforementioned evidence, there are also contradictory results, such as those of Kowal and Fortier (2000) and Reinboth and Duda (2006) who found no relationship between the task climate and competence need. Ahmadi et al. (2012) and Sánchez-Oliva et al. (2012) showed that a task climate predicts the three psychological needs, with greater force on the competence need, but the latter does not predict self-determined motivation (measured through an index). Recently, Monteiro et al. (2018) showed that the task climate is associated with psychological needs, being associated with autonomous motivation, while Rodrigues et al. (2020), in the physical education context, showed that the task climate is associated with each of the psychological needs.

### Motivation and Precompetitive Anxiety

The SDT postulates that motivation leads to different types of consequences of cognitive, emotional, and affective nature (Rodrigues et al., 2018). The most positive consequences would be produced by the more self-determined forms of motivation (i.e., autonomous motivation), while the most negative consequences would be produced by the less self-determined forms of motivation (i.e., controlled motivation and amotivation) (Ryan and Deci, 2017).

A maladaptive emotional consequence can be precompetitive anxiety, which is an immediate emotional state characterized by feelings of apprehension, tension, and nervousness generated by sports competition (Martens, 1977). This has been widely studied in the sports field because, together with self-confidence, they are psychological variables that considerably influence sports performance (León-Prados et al., 2014). The multidimensional theory of competitive state anxiety (Martens et al., 1990) proposes three components: somatic anxiety, which has to do with the physiological activation of the organism; cognitive anxiety, which is associated with cognitive representations of threat, uncertainty, and worry; and self-confidence, which is the degree of security that the athlete has about their ability to be successful in sports tasks. In the sports field, few studies have shown a positive association between more self-determined motivation and self-confidence before a competition (e.g., Zarauz and Ruiz-Juan, 2014; Pineda-Espejel et al., 2020) and no influence on the probability of presenting anxiety (e.g., Garcia-Mas et al., 2015; Pineda-Espejel et al., 2015, 2020), as well as a positive association between controlled regulations and precompetitive cognitive anxiety (e.g., Guillén and Álvarez-Malé, 2010); more studies reveal a positive association between amotivation and cognitive and somatic anxieties prior to competition (e.g., Amado et al., 2013; Zarauz and Ruiz-Juan, 2014; Pineda-Espejel et al., 2016, 2020).

### The Present Study

Sport coaches represent an integral part in the sports environment, interacting with athletes on a regular basis and having an impact on their motivation to participate in the sport (Smith et al., 2007); therefore, it is important to understand the contextual factors behind negative and positive experiences in high-performance sport.

Although the SDT assumes that the three psychological needs must be satisfied for self-determined motivation to occur (Deci and Ryan, 1985), the relatedness need in high-performance sport does not appear as a primary need (Hodge et al., 2009; Gené and Latinjak, 2014), while the satisfaction of autonomy and competence needs are of greater relevance in the mediation effect (e.g., Hodge et al., 2009). Some studies in the physical education context show that competence need is the most predictive factor on autonomous motivation (Rodrigues et al., 2020).

The antecedents mentioned here show contradictory evidence on the relationship between task climate, competence need, and its effect on self-determined motivation. In addition, there are some weaknesses, such as operationalizing motivation through an index. Howard et al. (2020) recommend using multidimensional methods for motivation instead of simplified scoring, in order to maximize the accuracy and predictive power of SDT's motivational conceptualization.

Relying on the horizontal organization of Vallerand's model (1997), which describes the sequence of social factors–psychological mediators–types of motivation–consequences, to our knowledge, no study has analyzed the complete Vallerand's sequence, including the impact of the adaptive social contexts created by the coach in satisfying competence need, regulation motivation, and possible consequences on negative emotional and cognitive states. Thus, the aim of this study was to analyze the motivational determinants of precompetitive anxiety in elite sport, considering the horizontal motivational sequence: adaptive social factors (i.e., task climate, autonomy support), competence need, types of motivation (i.e., autonomous motivation, controlled motivation, amotivation), and consequences (precompetitive anxiety and self-confidence). Also, this study analyzes the mediator role of competence and the motivational regulations on social factors and consequences.

**Figure 1:**
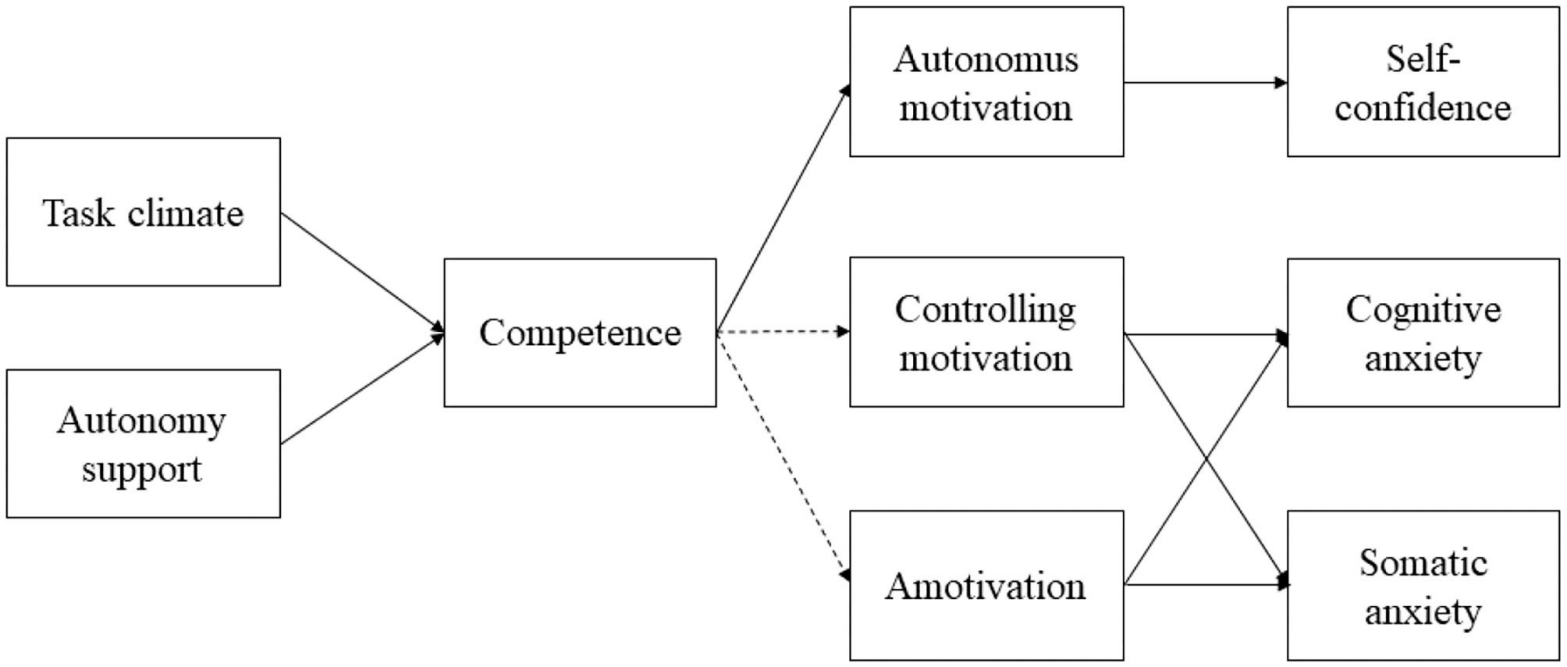
The hypothesized structural model. Solid lines represent positive effects and dashed lines represent negative effects.

The hypotheses of the study are as follows: more adaptive social factors are positively associated with competence need (hypothesis 1); competence need is positively associated with more self-determined motivational regulations and negatively with less self-determined motivation (hypothesis 2); and more self-determined motivation is positively associated with self-confidence, and less self-determined motivation is associated with anxiety (hypothesis 3). Additionally, competence need and motivational regulations will have a mediating effect within the model (Figure 1).

## Method

### Participants

Two hundred and seventeen athletes engaged in elite sport (112 men and 105 women) from ages 14 to 41 years (*M* = 22.83; SD = 5.68) participated in an intentional non-probabilistic sampling. They competed in the Central American and Caribbean Games Barranquilla 2018, in sports such as gymnastics (*n* = 14%), softball (*n* = 25%), synchronized swimming (*n* = 16%), swimming (*n* = 25%), and water polo (*n* = 10%), among others. They reported sports experience of 4.28 days per week (SD = 2.32) and an average length of training of 11.37 years (SD = 4.96). This sample was obtained from athletes who represent different countries such as Colombia (*n* = 7%), Costa Rica (*n* = 6%), Cuba (*n* = 12%), El Salvador (*n* = 6%), Guatemala (*n* = 18%), Mexico (*n* = 20%), Panama (*n* = 8%), Puerto Rico (*n* = 6%), and Venezuela (*n* = 17%).

### Instruments

The task-involved climate by the coach was measured with the Spanish version of the Perceived Motivational Climate in Sport Questionnaire (PMCSQ-2; López-Walle et al., 2011). The first-order subscale (task climate) has 11 items (e.g., “Athletes help each other to learn”), preceded by the initial phrase “In my training group...” The items are answered with a five-point Likert scale ranging from 1 (*never*) to 5 (*always*).

The coach's autonomy support for was measured with the short version of the Sports Climate Questionnaire (RS-SCQ; Balaguer et al., 2009), which has six items (e.g., “In my sport I feel comprehended by my coach”). These items are answered with a seven-point Likert scale ranging from 1 (*not true*) to 7 (*very true*).

The need for competence was measured with the perception of competence subscale in the Spanish version of the Basic Needs Satisfaction in Sport Scale (BNSSS; Pineda-Espejel et al., 2019). It consists of five items (e.g., “I am proficient in my sport”), which are answered with a seven-point Likert scale ranging from 1 (*not true*) to 7 (*very true*).

The motivational regulations were measured with the Spanish adaptation of the revised Scale of Motivation in Sports (SMS-II; Pineda-Espejel et al., 2016). This contains 17 items that answer the initial question “Why do you practice your sport?”. These items measure motivational regulations of the self-determination continuum: intrinsic motivation (e.g., “Because it is very interesting to learn how I can improve”), integrated (e.g., “Because playing sports shows the essence of who I am”), identified (e.g., “Because this is a way to develop myself”), introjected (e.g., “Because I wouldn't feel valuable or important if I didn't play it”), external regulations (e.g., “Because I think others would not value myself if I didn't”), and amotivation (e.g., “I'm not sure; I don't really think my place is in this sport”). The answers are collected on a seven-point Likert-type scale ranging from 1 (*does not correspond with me at all*) to 7 (*corresponds exactly with me*). According to SDT and previous sport research (e.g., Rodrigues et al., 2019), autonomous motivation (intrinsic motivation + integrated regulation + identified regulation) and controlled motivation (introjected + external regulations) variables were calculated and used as primary variables in the subsequent analysis.

Precompetitive anxiety and self-confidence perceptions were measured with the Revised Competitive State Anxiety Inventory-2 (CSAI-2R; Pineda-Espejel et al., 2014) with 17 items that respond to the initial question “How do you feel right now before the competition?”. These items are grouped into three factors: somatic anxiety (e.g., “I am very anxious”), cognitive anxiety (e.g., “I am worried about losing”), and self-confidence (e.g., “I feel confident”). Here, responses are collected on a four-point Likert scale ranging from 1 (*none*) to 4 (*a lot*).

### Procedure

This work was done in accordance with the ethical guidelines of the APA. Ethical approval of the Ethics Committee of the *Universidad Autónoma de Baja California* (registration reference: CE-UABC-2018:1149) was obtained prior to data collection. The study was supported by the vice principal of Medical Services and Doping Control of the Central American and Caribbean Games Barranquilla 2018. First, coaches and athletes were asked to participate; in the case of underage athletes, their coach or team delegate must present the informed consent to be part of the research. Participants were told that answering the questionnaires implied voluntary participation. They were also informed about data anonymity and confidentiality. Due to the logistics of the event, only 15 min was available to answer. The application of the instruments took place before the competition, within the same facilities, with the presence of the researchers to answer any doubts.

### Data Analysis

The data were analyzed at three moments: (1) reliability of the instruments, through McDonald's omega coefficient, where acceptable values are from 0.70 to 0.95 (Zinbard et al., 2005); (2) descriptive (mean and standard deviation) and Pearson's bivariate correlations between variables; and (3) structural equation models (SEM).

SEM were estimated from its two fundamental components: a measurement model and a structural model (Kline, 2015). For the first case, a confirmatory factor analysis (CFA), a model which confirms if the data fit to the proposed model, was conducted. For the second case, an analysis of structural equations using robust maximum likelihood with robust Satorra–Bentler corrections for standard and statistical errors was made (Finney and DiStefano, 2006). This analysis was carried out from compound scores, using the program LISREL 8.80 with the maximum likelihood estimation method. To evaluate the fit of the models, absolute indices were used, such as the relative chi-square (χ^2^*/df*) and the root mean square error of approximation (RMSEA) plus its 90% confidence interval (90% CI); incremental rates, utilizing the non-normed fit index (NNFI) and the comparative fit index (CFI), were assessed.

Regarding the χ^2^*/df* ratio, values between 2 and 3 are indicative of a good or acceptable data–model fit, respectively (Schermelleh-Engel et al., 2003). RMSEA values below 0.07 indicate a good model fit (Steiger, 2007). NNFI and CFI values ≥0.95 are presently recognized as indicative of a good fit (Hu and Bentler, 1999).

Considering theoretical and practical implications, serial mediation procedures were used to assess mediation effects. The SPSS PROCESS macro of Preacher and Hayes (2008) was used in the model 6 path analysis. The proposed model (i.e., model 6) ensures the control of the indirect effects for other estimated variables, allowing also independent mediator effect analysis and regression coefficients for each causal step of the indirect effects. Bias-corrected bootstrapped point estimates for the indirect effects of the independent variable on the dependent variable were estimated, considering standard errors and 95% confidence intervals. Bootstrap with 5,000 samples was used, and significant indirect effects were considered (at alfa = 0.05) if its 95% confidence intervals do not include zero (Hayes, 2013).

## Results

### Preliminary Analysis

Values obtained for all reliability coefficients were above 0.72 for the instruments, indicating strong internal consistency (Table 1). Means, standard derivations, and correlations for each measure are presented in Table 1. Correlations between study variables were consistent with expected associations based on the proposed model. For example, the task climate as well as the autonomy support was positively related to competence, and competence was positively associated with autonomous motivation and negatively with controlled motivation and amotivation. Autonomous motivation was positively related to self-confidence; controlled motivation and amotivation were negatively related to self-confidence and conversely with somatic and cognitive anxieties.

**Table 1:** Reliability, descriptive, and correlation matrix to the study variables.

	**Variable**	**1**	**2**	**3**	**4**	**5**	**6**	**7**	**8**	**9**
1	Task climate	0.87								
2	Autonomy support	0.42^**^	0.94							
3	Competence	0.37^**^	0.27^**^	0.82						
4	Autonomous motivation	0.26^**^	0.18^**^	0.31^**^	0.89					
5	Controlled motivation	−0.7	0.02	−0.17^**^	0.15^**^	0.75				
6	Amotivation	−0.23^**^	−0.17^**^	−0.28^**^	−0.34^**^	0.49^**^	0.72			
7	Self-confidence	0.30^**^	0.24^**^	0.31^**^	0.35^**^	−0.21^**^	−0.24^**^	0.80		
8	Cognitive anxiety	−0.08	−0.09	−0.12	−0.02	0.14^**^	0.27^**^	−0.23^**^	0.85	
9	Somatic anxiety	−0.04	−0.07	−0.13	−0.05	0.20^**^	0.26^**^	−0.20^**^	0.59^**^	0.84
*M*		4.07	4.93	6.01	5.51	2.88	2.22	3.18	1.93	2.14
SD		0.65	1.42	0.88	1.17	1.37	1.40	0.64	0.65	0.77
Range		1–5	1–7	1–7	1–7	1–7	1–7	1–4	1–4	1–4

** *p < 0.01; the diagonal shows McDonald's omega values*.

### Confirmatory Factorial Analysis and Structural Equation Model

The process of validating the measurement model through CFA determined that the observed variables were indeed good indicators of the latent variables. The measurement model of each scale in the current study provided adequate fit (Table 2).

**Table 2:** Fit indices of the measurement models.

**Instrument**	**χ^**2**^**	***df***	**RMSEA (90% CI)**	**CFI**	**NNFI**
Task climate subscale (PMCSQ-2)	632.11	229	0.056 (0.043–0.075)	0.90	0.91
RS-SCQ	19.63	22	0.067 (0.050–0.090)	0.99	0.98
Competence subscale (BNSSS)	38.53	13	0.052 (0.040–0.070)	0.91	0.92
SMS-II	306.55	120	0.079 (0.067–0.093)	0.957	0.962
CSAI-2R	206.39	116	0.062 (0.048–0.075)	0.978	0.974

**Figure 2:**
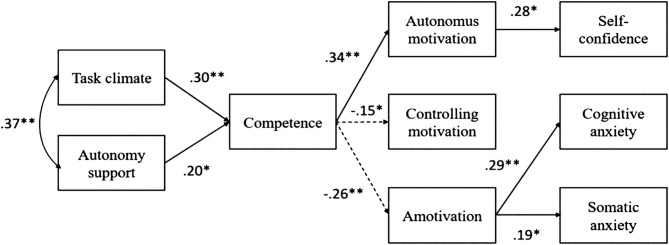
Standardized solution of the hypothesized structural model of mediation. *p < 0.05; **p < 0.01.

The structural model provided a good fit: χ^2^/*df* = 1.62; RMSEA = 0.053 (90% CI = 0.01–0.08); CFI = 0.97; NNFI = 0.94. As can be seen in Figure 2, there were positive and significant effects from the task climate and autonomy support toward competence need, from competence need to autonomous motivation, and from the latter toward self-confidence. In addition, there were significant negative effects of competence need toward controlled motivation and amotivation. Finally, amotivation was positively related to both anxieties (i.e., somatic and cognitive). The significant paths (*p* < 0.05) are presented in Figure 2.

### Multiple Serial Mediation

**Figure 3:**
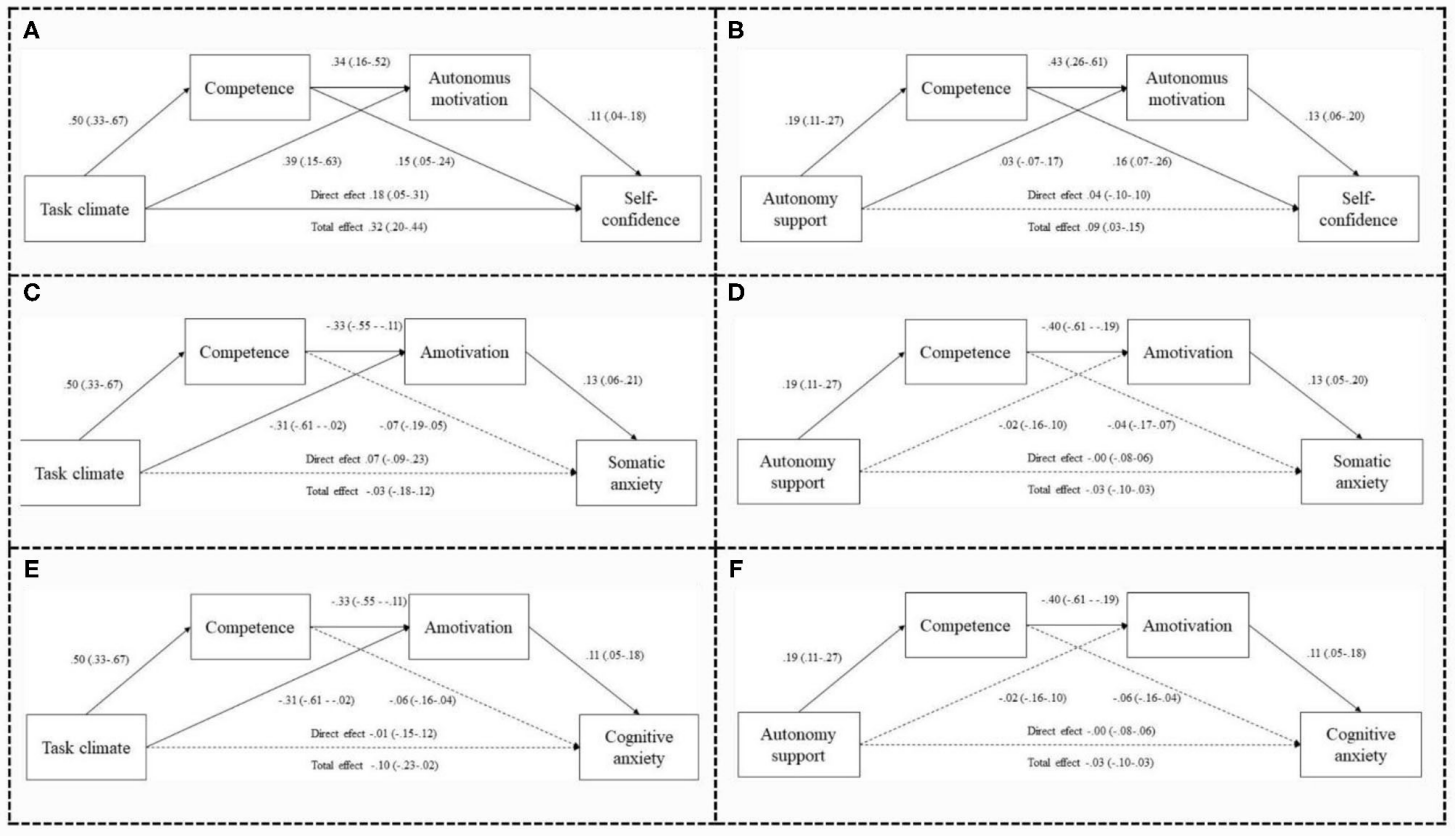
Serial mediation models. 95% CI estimate indicates a significant indirect effect. Significant effect—continuous lines; non-significant effect—dashed lines. **(A–F)** Refers to each of the serial mediation models tested.

From the SEM results, six serial mediation effects were analyzed: three ranging from task climate to self-confidence, somatic anxiety, and cognitive anxiety, respectively. Additionally, there were three effects ranging from autonomy support to self-confidence, somatic anxiety, and cognitive anxiety, respectively. In Figure 3, the mediating effects of competence need and motivational regulations in the relationship between social factors and consequences of precompetitive self-confidence and anxiety are presented.

The task climate, in the mediation models, revealed a positive and significant association with competence and autonomous motivation (Figure 3A), and in the other two models of mediation (Figures 3C,E), there were a positive association with competence and a negative association with amotivation. Competence maintained a positive association with autonomous motivation (Figure 3A), and in the other two models, competence had a negative association with amotivation (Figures 3C,E). On the other hand, autonomous motivation and competence had significant positive direct effects on self-confidence (Figure 3A), and amotivation had significant positive direct effects with somatic and cognitive anxieties (Figures 3C,E). In the direct effect analysis, the model that goes from task climate to self-confidence showed a total mediation (Figure 3A).

Autonomy support, in the mediation models, revealed a positive and significant association with competence (Figures 3B,D,F) and with autonomous motivation (Figure 3B). Competence was positively and significantly associated with autonomous motivation (Figure 3B) and negatively with amotivation (Figures 3D,F). Autonomous motivation was positively and significantly associated with self-confidence (Figure 3B), and amotivation was associated with somatic and cognitive anxieties (Figures 3D,F). In the direct effect analysis, there were no significant effects from autonomy support to consequences.

The comparison between mediators was made for each proposed mediation. Although the indirect effects are statistically significant, in statistical terms, the sequences presented in Table 3 were superior.

**Table 3:** Superior combined indirect effects after contrast of mediators in the tested sequences.

**Significantly higher sequences**	**Coefficient (95% CI)**
Task climate → autonomous motivation → self-confidence	0.04 (0.01 to 0.08)
Task climate → amotivation → cognitive anxiety	−0.03 (−0.09 to −0.01)
Task climate → amotivation → somatic anxiety	−0.04 (−0.10 to −0.01)
Autonomy support → competence → self-confidence	0.03 (0.01 to 0.05)
Autonomy support → competence → amotivation → cognitive anxiety	−0.01 (−0.02 to −0.01)
Autonomy support → competence → amotivation → somatic anxiety	−0.01 (−0.02 to −0.01)

## Discussion

Most of the stated hypotheses were confirmed, and the results were aligned with the AGT (Deci and Ryan, 1985) and SDT (Nicholls, 1984, 1989) predictions. In this case, the athlete's perception that his coach generates high task-involved climate and autonomy support helps the athlete to feel effective when interacting with his training environment and when executing techniques and sport skills. This agrees with other studies in the field sport (e.g., Sarrazin et al., 2002; Ommundsen et al., 2006, 2010; Almagro et al., 2011; Ahmadi et al., 2012; Balaguer et al., 2012; Isoard-Gautheur et al., 2012; Pope and Wilson, 2012; Sánchez-Oliva et al., 2012) and disagrees with other studies (e.g., Kowal and Fortier, 2000; Reinboth and Duda, 2006).

The result above answers to the fact that when individual criteria are used to evaluate and reward the performance, focusing more on aspects of self-improvement and effort, people feel more competent and less threatened because the results evaluated are more controllable (Ames, 1992). Meanwhile, the autonomy support offers the athlete opportunities to learn and express sport abilities, and this helps to feel capable of achieving goals and the desired consequences (Deci and Ryan, 2002). It supports that the motivational climate that the trainer creates is an important environmental factor affecting the need for competence.

The next part of the sequence explains that when athletes feel effective in sport abilities and skills, then they participate in a sport because they experienced actions emanating from themselves (Ryan and Connell, 1989) and also because they are interested and experienced enjoyment from the activity (Amorose and Anderson-Butcher, 2007). This agrees with other studies (e.g., Frederick and Ryan, 1993; Sarrazin et al., 2002; Ommundsen et al., 2010; Pope and Wilson, 2012), but does not agree with Ahmadi et al. (2012), Isoard-Gautheur et al. (2012), and Sánchez-Oliva et al. (2012) who did not obtain significant associations between these variables, which may be due to the different study designs and the operationalization of the variables. The result supports the fact that the need for competence contributes to the facilitation of the internalization of behavior and the consequent self-regulation of extrinsically motivated activities (Ryan and Deci, 2000).

Moreover, the need for competence opposes the high-performing athlete from practicing due to internal and/or external pressures and external control (Ryan and Connell, 1989; Deci and Ryan, 2002) or from practicing without conviction. In contrast, the feelings for competence are associated with intrinsic motivation. This result is partially consistent with other studies (e.g., Pope and Wilson, 2012), and it opposes the positive association between these variables offered by Rodrigues et al. (2019).

The last part of the model explains how motivation is associated with anxiety consequences. This study replicates that autonomous motivation is associated with more adaptive outcomes, since autonomous motivation favors athletes to have a high degree of certainty that they can succeed in their sport and, thus, intensify their efforts to achieve their goals. Conversely, if athletes are not motivated to practice their sport, then, before a competition, they will have high physiological activation, as well as uncertainty and concern, according to other studies (Amado et al., 2013; Zarauz and Ruiz-Juan, 2014; Pineda-Espejel et al., 2016, 2020). This may be because unmotivated athletes feel incompetent, leading to uncertainty about their performance and expected results, so they focus on their mistakes, and these doubts create anxiety (Weinberg and Gould, 2010), affecting the quality of attention (Ries et al., 2012).

From the serial mediation analysis, the mediators show similar trends as in the SEM, although different effects are observed from both adaptive social factors. All models that start from the task climate had significant direct and indirect effects on the consequences of precompetitive anxiety and self-confidence. For models that start from autonomy support, all the indirect effects on the consequences were significant, but not for all the direct effects. In sum, task climate has a greater effect on self-confidence through autonomous motivation in isolation, than it does through the effects of the two mediators operating in series. Autonomy support has a greater effect on self-confidence through the athlete's competition need. In addition, when a task climate is perceived, there is less somatic and cognitive anxiety through reducing the athlete's amotivation. On the other hand, with autonomy support, there is less somatic and cognitive anxiety when the competition need of the athletes is satisfied and there is also less amotivation for the sport.

The current study has a number of strengths, including the population characteristics, exploration of the direct and indirect effects of the models, and capturing of regulation-specific effects using more comprehensive scoring practices as they maximize construct-relevant information. The present study contributed to the dissemination of knowledge in the elite sports context and corroborated the assumptions of Ryan (1995), which stated that research carried out with SDT should be done in a specific context.

From a practical point of view, when coaches, of athletes engaged in elite sport, promote a task climate or autonomy support, they satisfy the athletes' need to demonstrate their own capabilities. This is important because athletes will have a high degree of assurance of success in competition, as well as less uncertainty or worry. A model of motivation that integrates conceptualizations of achievement and support could be an important tool that promotes adaptive outcomes. Coaches who promote a task-involving climate and autonomy support improve the competence need satisfaction of athletes. These climates will facilitate the regulation of their behavior toward autonomous motivation, with positive results in the athletes' self-confidence. Subsequently, sport psychologists can design intervention alternatives for coaches to set goals, to direct training, to model attitudes, to create strategies, or to manage competition situations in a more positive way, for example, by allowing to select and choose the results to achieve (Deci and Ryan, 1987).

This study has limitations related to the characteristics of the sample (high-performance athletes, chronological age, and cultural diversity) and to the transversal design, which only allows the adjustment of the model in a given time slot. Therefore, the results cannot be generalized to populations with other characteristics nor can be used to establish solid causal relationships. In addition, the data were collected before the competition, which influenced the anxiety states. Although the current results point to a possible role of the social context on anxiety and self-confidence, future studies are suggested that integrate the major social environmental dimensions emphasized within AGT and SDT (e.g., ego climate, thwarting interpersonal behaviors), and relatedness and autonomy needs are to be included to enrich the model following the AGT and its relationship with the constructs of the SDT.

## Conclusion

Overall, our hypotheses were supported by the present results. As was empirically demonstrated, task climate and autonomy support are associated with more adaptive patterns, favoring self-confidence, while at the same time being opposed to presenting anxiety states prior to a competition in high-performance sports. In addition, there is a total mediation of competence need and autonomous motivation on the association between task climate and self-confidence.

## Data Availability Statement

The raw data supporting the conclusions of this article will be made available by the authors, without undue reservation.

## Ethics Statement

The studies involving human participants were reviewed and approved by Ethics Committee of the Universidad Autónoma de Baja California. Written informed consent to participate in this study was provided by the participants' legal guardian/next of kin.

## Author Contributions

All authors listed have made a substantial, direct and intellectual contribution to the work, and approved it for publication.

## Conflict of Interest

The authors declare that the research was conducted in the absence of any commercial or financial relationships that could be construed as a potential conflict of interest.
